# Effect of facial expressions on student’s comprehension recognition in virtual educational environments

**DOI:** 10.1186/2193-1801-2-455

**Published:** 2013-09-12

**Authors:** Mohamed Sathik, Sofia G Jonathan

**Affiliations:** Sadakathullah Appa College, Tirunelveli, India; Research and Development Centre, Bharathiar University, Coimbatore, India

**Keywords:** Facial expression, Non-verbal communication, Virtual classroom, Action units, Comprehension

## Abstract

The scope of this research is to examine whether facial expression of the students is a tool for the lecturer to interpret comprehension level of students in virtual classroom and also to identify the impact of facial expressions during lecture and the level of comprehension shown by these expressions. Our goal is to identify physical behaviours of the face that are linked to emotional states, and then to identify how these emotional states are linked to student’s comprehension. In this work, the effectiveness of a student’s facial expressions in non-verbal communication in a virtual pedagogical environment was investigated first. Next, the specific elements of learner’s behaviour for the different emotional states and the relevant facial expressions signaled by the action units were interpreted. Finally, it focused on finding the impact of the relevant facial expression on the student’s comprehension. Experimentation was done through survey, which involves quantitative observations of the lecturers in the classroom in which the behaviours of students were recorded and statistically analyzed. The result shows that facial expression is the most frequently used nonverbal communication mode by the students in the virtual classroom and facial expressions of the students are significantly correlated to their emotions which helps to recognize their comprehension towards the lecture.

## Background

Today’s learning community focus on the vision of faculty and students working collaboratively towards deep, meaningful, high quality learning. The achievements of digital communication lead learning communities into a new dimension. By means of communication technologies using different types of learning tools, spaces and forms of interaction virtual learning communities are emerging for many universities and colleges. Distance education enables many learners the opportunity to experience virtual learning environments. As the World Wide Web becomes an increasingly more popular medium for instructional delivery of distance education, the vision of faculty and students working collaboratively in a virtual learning community is becoming a reality. The explosion of the knowledge age has changed the context of what is learnt and how it is learnt to the concept of virtual classrooms was a manifestation of this knowledge revolution.

There is an increase in virtual schools worldwide (Theonas et al. 2007) and it is suggested that education mediated by computer is considered very important for the future. However, a major drawback of present virtual schools is the large number of drop out students. There are many areas that need to be studied and improved concerning the effectiveness of the virtual lectures, and it is believed that more studies are needed in order to establish the ‘ingredients’ in an educational virtual environment that can motivate the students. In particular, different researchers suggest that the visual representation of the participant in a virtual environment, possibly in a virtual classroom, increases the potential for person-to-person collaboration and interaction. This research specifically focused on the facial expressions of the students and the role they play during the virtual lecture. The aim was to identify the level of comprehension shown by these expressions which helps the virtual lecturer to improve their teaching style accordingly that keeps the students interested and enthusiastic during the virtual lectures.

### Virtual classroom

A virtual classroom (Marco Van Der 2005) is the use of video, audio and other technology to simulate the traditional class and learning environment as closely as possible. Virtual environments may be used for a plethora of pedagogical purposes (Jelfs & Colbourn 2002) such as distance education. There is an increase in virtual schools worldwide as education mediated by computer is considered very important for the future (Russell & Holkner 2000). Virtual Education (Kurbel 2001) refers to instruction in a learning environment where teacher and student are separated by time or space, or both, and the teacher provides course content through course management applications, multimedia resources, the Internet, videoconferencing, etc. Students receive the content and communicate with the teacher via the same technologies.

The physical classroom is a physical room that must be visited at an appropriate time in order to participate in, while a virtual classroom is not physically accessed. This difference makes a virtual classroom, available to many, adaptable and flexible because of its non- physical location (Oakes 2002). Virtual classrooms tend to encourage collaborative learning (Taxen & Naeve 2002), because more information and knowledge can be gained through the interaction and involvement with virtual class members than solely through the reception of information from an instructor.

### Virtual class room communication

A real classroom enables live face-to-face communication (Mohamed Sathik & Sofia 2011); many virtual classrooms aim to implement this by having regularly scheduled chat room with video conferencing interactions where students can interact with each other and the lecturer as they would in a real classroom. Hence, the classroom communication in virtual classroom is analogous to the communication in real classroom. In virtual classrooms, synchronous communication is used for learning and teaching, it has been referred as a channel of communication, which learners use to communicate with fellow class members and their lecturer (Burbles 2004; Huloria). In the classroom, lecturers and students--both consciously and unconsciously--send and receive nonverbal cues several hundred times a day (Mohamed Sathik & Sofia 2011). Lecturers should be conscious of nonverbal communication in the classroom for two basic reasons: to become better receivers of student’s messages and to gain the ability to send positive signals that support student’s learning while simultaneously becoming more skilled at avoiding negative signals that suppress their learning. It is just as important for lecturers to be good nonverbal communication senders as it is for them to be good receivers.

### Facial expressions

Teacher student Interaction plays a vital role in any classroom environment (Mohamed Sathik & Sofia 2011). The impact due to communication of the face is so powerful in interaction. Faces are rich in information about individual identity, and also about mood and mental state, being accessible windows into the mechanisms governing our emotions. Studies reveal that the most expressive way humans display emotions is through facial expressions. Facial expressions are the primary source of information, next to words, in determining an individual’s internal feelings.

All people thus certainly Lecturers and students use facial expressions to form impressions of another. A study had revealed that the facial expressions of the lecturers kept the students motivated and interested during the lectures (Toby et al. 2008). A Lecturer can also use student’s facial expressions as valuable sources of feedback. While delivering a lecture, a Lecturer should use student’s expressions to determine whether or not to slow down, speed up, or in some other way modify his presentation. The basic strategy of optimizing the classroom behavior is that the teachers must have the capability to feel student’s minds changing; they must be good at observing student’s facial expression, every action and movement. This helps the Lecturers to understand their own weakness and to change it.

Lecturers should be highly skilled in understanding the emotions in order to identify the comprehension of the students from their facial expressions itself*.* If the Lecturers are not able to identify the significance in the facial expressions it will undermine the understanding of the students, thereby, create negative impact on student’s learning.

Momentary expressions that signal emotions include muscle movements such as raising the eyebrows, wrinkling the forehead, rolling the eyes or curling the lip (Resmana Lim & Reinders 2000). When students are feeling uncomfortable, they may have lowered brow, drawn together brow, horizontal or vertical forehead wrinkles, and have a hard time in maintaining eye contact. To be a good receiver of student messages, a lecturer must be familiar to many of the subtle nonverbal cues that their students send.

Studies have evaluated that student’s emotional states are expressed with specific behaviours that can be automatically detected (Toby et al. 2008). Detecting facial landmarks (such as position of Forehead, eyes, nose, mouth, etc.) play an important role in face recognition systems (Russell & Holkner 2000) as they act as the action units of the face, which determine the denotation behind the expressions (Jain 1999) indicated by them. Recognition of emotions from facial expressions involves the task of categorizing active and spontaneous facial expressions to extract information about the underlying emotional states.

In this study, the main hypothesis of the first step proposed that facial expression is the widely used nonverbal communication mode by the students in the classroom which in turn helps the lecturers to identify the comprehension of the students. The second step proposed that the facial expressions through the action units (Eyes, Mouth, Eyebrows and Forehead) help the lecturers to identify the involvement and comprehension of the students in the classroom during the lecture. The third step proposed that the student’s expressions are significantly correlated to their emotions that in turn identify their level of comprehension. The significance of the study was statistically interpreted.

The remainder of this paper is organized as follows. The experimental results are discussed in Section 'Experimental Results'. Conclusion and directions for future work are briefly covered in Section 'Conclusion'. The methods adopted in this paper are presented in Section 'Methods'.

## Experimental results

Facial Expressions that signal emotions include muscle movements such as raising eyebrows, wrinkling the forehead, rolling the eyes or curling the lip (Perry and Bruce 2004). Therefore the action units of face such as eyes, mouth, eyebrow and forehead are the emotion indicators. Here we analyzed whether the emotion of the students with respect to comprehension are indicated through expressions of facial action units. Experimentation was done with survey and analysis through SPSS and MS-Excel. Sample images to represent the different expressions are taken from JAFE and YALE Face databases.

### Analysis: non-verbal communication

**Figure 1:**
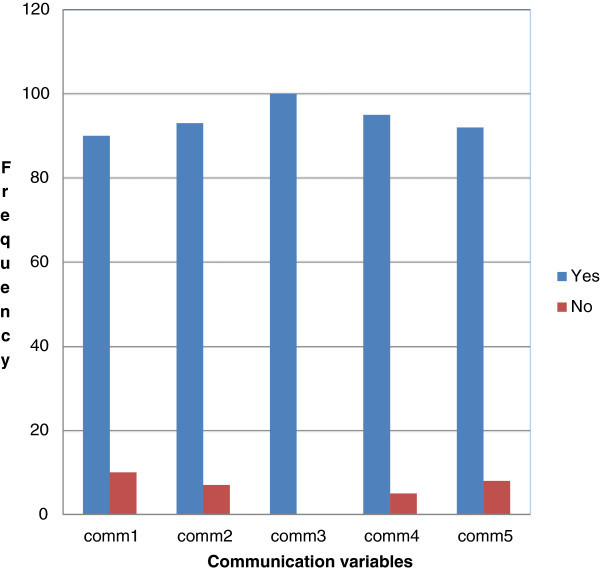
**Frequency of communication variables.**

The first step of the experiment proposed that the facial expression is the widely used nonverbal communication mode by the students in the classroom which in turn helps the lecturers to identify the comprehension of the students. To prove the effectiveness, student’s expressions that are used for non-verbal communication in virtual pedagogical environments was investigated first. It was measured using five communication variables. Figure 1 represents the frequency percent of communication variables.

Communication variables symbolize the following information.

- Comm1: Non-‘verbal communication is the most frequently used communication skill in the classroom during the lecture.
- Comm2: Facial expressions of the student affect the teaching process.
- Comm3: Lecturer can identify the comprehension of students in classroom using their suitable facial expression.
- Comm4: When the students are not able to follow the lecture, they use facial expressions to express it.
- Comm5: When the student is able to understand the lecture, they use facial expressions to express it.

Figure 1, depicts that frequency percent of ‘yes’ is very high when compared to the frequency percent of ‘No’ which infers that facial expression is the widely used nonverbal communication mode by the students in the classroom which in turn helps the lecturers to identify the comprehension of the students.

Commonly used Nonverbal communication modes are ranked by the observers and the orders of merit given by them were converted into ranks using Henry Garrett Ranking Technique. In this technique percentage position of the ranks are calculated using Equation 1.1

Where,

R_ij_ – Rank given for ith item by jith individual.

N_j_ – Number of items ranked by jth individual.

The percentage position of each rank thus obtained was converted into scores by referring Garrett Ranking table. Then the number of respondents of each rank for a particular nonverbal communication mode is multiplied with its corresponding score. Then Mean score or Weighted score is calculated by finding the summation of the products. Finally the mean scores or the weighted scores are ordered to get the ranks which are given in Table 1.

**Table 1:** **Ranking of nonverbal communication modes**

Mode of nonverbal communication	Mean score/Weighted score	***Rank***
Facial expression	9536	**I**
Hands	8668	**IV**
Gestures	8882	**III**
Body language	8943	**II**

**Figure 2:**
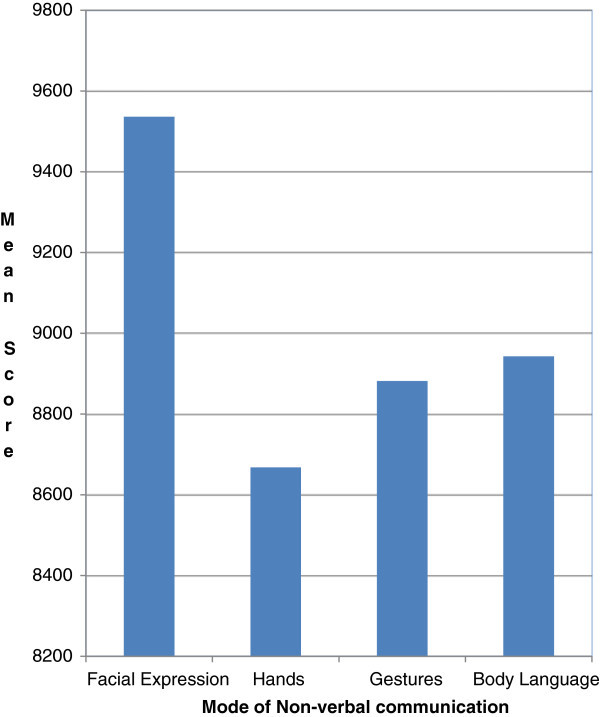
**Comparison of non-verbal communication modes.**

Table 1 clearly indicates that Facial expression is ranked high by the lectures for the frequent and effective mode of Nonverbal communication in the classroom during the lecture which helps them to interpret the level of comprehension of the students. Scores are pictorially represented in Figure 2.

### Analysis: action units

The second step of the experiment proposed that the facial expressions of the action units (Eyes, Mouth, Eyebrows and Forehead) the lecturers to identify the involvement and comprehension of the students in the classroom during the lecture. The analysis made to check the effectiveness of the interpretation of student’s comprehension through facial expressions signaled by action units.

It is important to check whether facial expressions of the students have impact on their comprehension of the lecture. In order to examine the degree of association between these two variables, Pearson’s correlation are calculated, followed by a *T*-test to determine the statistical significance of this. Table 2 shows the mean scores of the expressions of the action units.

**Table 2:** **Mean score of facial expressions**

Facial expressions	Mean score
Eye shrink	3.670
Eye enlarge	4.310
Mouth widen	3.590
Lips curled	3.330
Eyebrow raised	4.040
Eyebrow lowered	3.300
Forehead wrinkle	3.390

**Figure 3:**
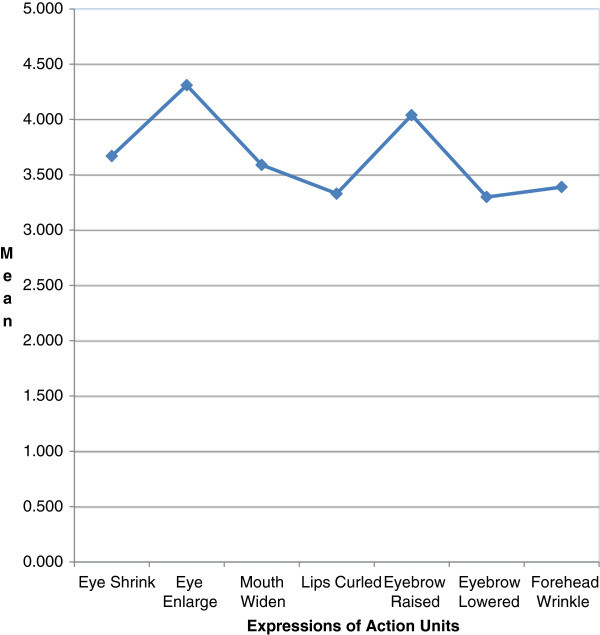
**Comparison of mean scores of action units in expressing students emotions.**

Figure 3 shows that the facial expressions indicated by the action units for expressing their comprehension of the lecture is above the average level ( >3).

Thus, the presence of positive or negative emotions of the students can help to recognize their comprehension towards the lecture with the help of their corresponding facial expressions. Other expressions are below the average which confirms the lecturer is in unpredictable state.

The significance of the role of the action units in expressing the comprehension is tested using *T*-test and the observation is given in Table 3.

**Table 3:** **Significance of action units**

Variable	Mean	T	P
Action units	25.2600	23.278	<.001**

** Correlation is significant at 1% level.

Table 3, shows that the measured facial expressions indicated by the action units are above the average level (25.2600 > 15) where 15 is the Testvalue. T value shows there is correlation between the students comprehension and the facial expressions. Thus the facial expressions (Eye shrink, Eye Enlarge, Mouth Widen, Lip curl, Eyebrow Raise, Eyebrow Lowered, Forehead Wrinkles) of the action units (Eyes, Mouth, Eyebrows and Forehead) helps the lecturers to identify the involvement and comprehension of the students in the classroom during the lecture.

### Analysis: facial expressions

The third step proposed that the student’s expressions are significantly correlated with their emotions which in turn identify their level of comprehension. Hence, we evaluated Pearson’s correlation between the facial expressions, and the student’s emotional feelings. Correlation and Standard deviation were computed between positive emotions (comprehension) and positive expressions. Then we calculated the correlation between negative emotions (incomprehension) and negative expressions.

Table 4, significantly shows that student’s expressions are positively correlated to their emotions which in turn identify their level of comprehension.

**Table 4:** **Correlation between expressions and emotions**

Expression – Emotion	Pearson correlation coefficient
Positive –Positive	0.571452
Negative –Negative	0.523

**Figure 4:**
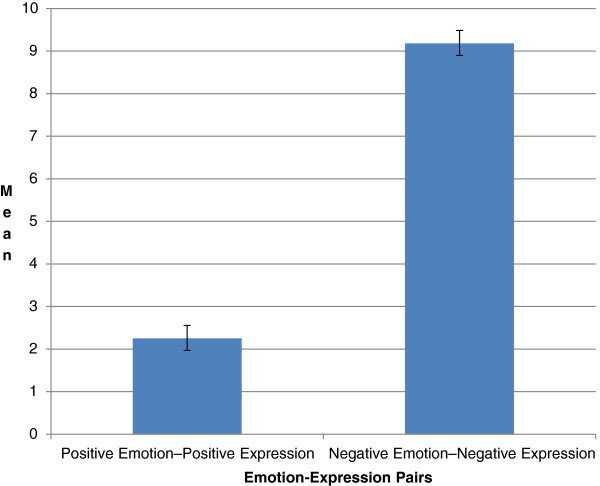
**Representation of standard error mean.**

The significance of the association between emotions and expressions are tested using *T*-test and the observation is given in Table 5. Standard Error Bar in Figure 4 clearly depicts that the error value is very negligible.

**Table 5:** **Paired samples test**

Pairs	Mean	Standard deviation	Standard error mean
Positive emotion – Positive expression	2.2500	3.03640	.30364
Negative emotion – Negative expression	9.1800	2.80469	.28047

The above statistical analysis strongly suggests that facial expression is the most frequently used nonverbal communication mode used by the student in the classroom and student’s expressions are significantly correlated to their emotions, where which can help to recognize their comprehension in the lecture. In particular, the more expressive the student was more the lecturer recognizes the comprehension of the students.

## Conclusion

Recent research documents notify that understanding emotional expressions play an important role in the development and maintenance of communal relationships. By accurately interpreting other’s emotions, one can obtain valuable information. All people thus certainly teachers and students use facial expressions to form impressions of another. This paper statistically proved that facial expressions of the students are the most used nonverbal communication mode in the classroom and student’s expressions are significantly correlated to their emotions which can help to recognize their comprehension towards the lecture. Hence this research concludes that Facial Expression plays a vital role in identification of Emotions and Comprehension of the students who are geographically distributed in the virtual classrooms as the classroom communication in virtual classroom is analogous to the communication in real classroom. The effectiveness of this method will be improved by correlating more features from different action units of the face which would improve the recognition rate. The limitation of the method is that if the visualization of the student is not available in the virtual lecture, facial expressions cannot be used a tool by the virtual lecturer to interpret student’s comprehension.

## Methods

In this research, a study was conducted for observing the facial expressions of the students in academic lecture-environments. The scope of this research is to examine whether facial expression of the students is a tool for the lecturer to interpret comprehension level of students in virtual classroom and also to identify the impact of facial expressions during lecture and the level of comprehension shown by these expressions.

In order to collect data for the study, survey is taken using stratified sampling technique with a questionnaire. Questionnaire is prepared based on the information collected by interviewing 80 undergraduate and postgraduate students in the age group of 18–22. It was given to 100 Lecturers both male and female in the age group of 35–58; whose experience is more than 10 years and their responses were collected. Further information or clarification was given to Lecturers orally, if necessary, while they were completing the questionnaires. This method of data collection ensures a high response rate, accurate sampling and a minimum of researcher bias, while giving the benefit of a degree of personal contact. The questionnaire was formatted with closed-ended (Yes/No), Liker scale (Strongly Agree, Agree, Undecided, Disagree, Strongly Disagree) and Ranking (Rank I to IV) questions. This is mainly because the use of a questionnaire enables the collection of more information, and therefore a more representative description of a situation, than interviews. Furthermore, the use of Liker scale is a very widely used technique for attitude measurement, and people often enjoy completing a scale of this kind (Oppenheim, 1992; Foddy, 1993).

This questionnaire focused on the role of facial expressions in non-verbal communication. It ranks the order in which the lecturer interprets the level of comprehension in the classroom through various nonverbal communication modes. The communication modes considered are Facial expressions, Gesture, Hand Movement and Body Language. It also measures the frequency of the expressions exhibited by the action units (Eyes, Mouth, Eyebrow and Forehead) of face for the purpose of communication. Finally, how the expressions were correlated with the emotions of the student’s is analyzed. Here Positive expressions are analyzed with positive emotion (Comprehension) and negative expressions are analyzed with negative emotion (Incomprehension). It was concluded from the student’s interview and previous studies that students convey positive emotions when they:

- Understand the lecture
- Are satisfied with the lecture
- Are able to grasp the ideas given by the lecturer.
- Want to reflect positive response to the lecture.

**Figure 5:**
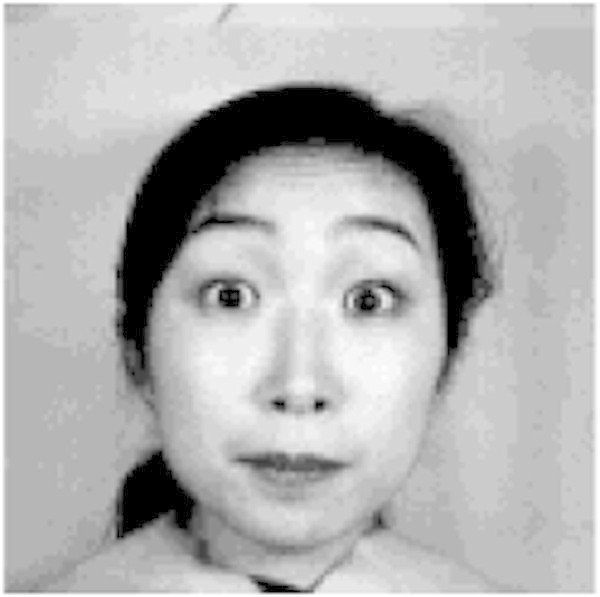
**Positive expression.**

Positive emotions are expressed by Eyes opening wide and raising Eyebrows as shown in Figure 5 to express their comprehension on the lecture.

Students convey negative feelings when they:

- Don’t understand the lecture.
- Want the lecturer to repeat once again.
- Try to seek the help of the lecturer.
- Are not able to cope up with the speed of the lecturer.
- Are in confused state.

**Figure 6:**
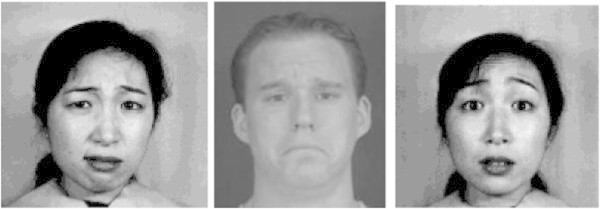
**Negative expression.**

Negative emotion are expressed by shrinking eyes with lowering eyebrows and wrinkles on forehead, eyebrows raised and eyes enlarged, curling lips as shown in Figure 6 to represent their incomprehension on the lecture.

**Figure 7:**
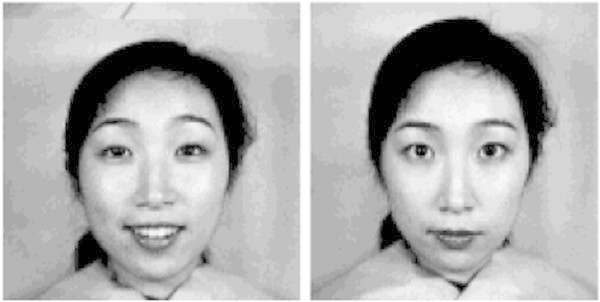
**Undecided emotional states.**

Lecturers will be in undecided state when the emotional states of the students are neutral, smile etc. as shown in Figure 7, which confuses the lecturer between comprehension and incomprehension states of the students.

## Authors’ information

Dr.M.Mohamed Sathik has been working as Principal of Sadakathullah Appa College, Tirunelveli. He has completed M.Phil., and Ph.D. in Computer Science in Manonmaniam Sundaranar University, Tirunelveli. He has added credit to his academic record by adding up the degrees M.Tech.,M.S(Psychology) and M.B.A. He is pursuing Post Doctoral Degree in Computer Science. He has also involved himself in various academic activities. He has attended many national and international seminars, conferences and presented numerous research papers. His had his publications in many International journals. He has guided more than 40 research scholars and published two books. He is a member of curriculum development committee of various universities and autonomous colleges of Tamilnadu, India. His areas of specialization are Virtual Reality, Image Processing and Sensor Networks.

G. Sofia has been working as an Assistant Professor in the Department of Computer Science, Lady Doak College, Madurai. She has completed M.C.A in Manonmaniam Sundaranar University, Tirunelveli and M.Phil., Computer Science in Mother Teresa Women’s University, Kodaikanal. She is pursuing Ph.D in Bharathiar University, Coimbatore under the valuable guidance of Dr.M.Mohamed Sathik. She has presented many papers in National and International conferences and published in 4 International journals. Her areas of specialization are Image Processing and Virtual Reality.
